# POH1/Rpn11/PSMD14: a journey from basic research in fission yeast to a prognostic marker and a druggable target in cancer cells

**DOI:** 10.1038/s41416-022-01829-z

**Published:** 2022-05-02

**Authors:** Vito Spataro, Antoine Buetti-Dinh

**Affiliations:** 1 Service of Medical Oncology, Oncology Institute of Southern Switzerland (IOSI), Ospedale San Giovanni, Via Gallino, 6500 Bellinzona, Switzerland; 2 Institute of Microbiology, Department of Environmental Constructions and Design, University of Applied Sciences and Arts of Southern Switzerland (SUPSI), via Mirasole 22a, 6500 Bellinzona, Switzerland; 3 Swiss Institute of Bioinformatics, Quartier Sorge, Batiment Genopode, 1015 Lausanne, Switzerland

**Keywords:** Molecular medicine, Oncogenes, Drug development

## Abstract

POH1/Rpn11/PSMD14 is a highly conserved protein in eukaryotes from unicellular organisms to human and has a crucial role in cellular homoeostasis. It is a subunit of the regulatory particle of the proteasome, where it acts as an intrinsic deubiquitinase removing polyubiquitin chains from substrate proteins. This function is not only coupled to the translocation of substrates into the core of the proteasome and their subsequent degradation but also, in some instances, to the stabilisation of ubiquitinated proteins through their deubiquitination. POH1 was initially discovered as a functional homologue of the fission yeast gene pad1^+^, which confers drug resistance when overexpressed. In translational studies, expression of POH1 has been found to be increased in several tumour types relative to normal adjacent tissue and to correlate with tumour progression, higher tumour grade, decreased sensitivity to cytotoxic drugs and poor prognosis. Proteasome inhibitors targeting the core particle of the proteasome are highly active in the treatment of myeloma, and recently developed POH1 inhibitors, such as capzimin and thiolutin, have shown promising anticancer activity in cell lines of solid tumours and leukaemia. Here we give an overview of POH1 function in the cell, of its potential role in oncogenesis and of recent progress in developing POH1-targeting drugs.

## Introduction

Fundamental mechanisms that are highly conserved in evolution can be discovered and studied in unicellular eukaryotes, such as yeasts. The fission yeast (*Schizosaccharomyces pombe*) model has led to important advances in studies of cell cycle and cell division [1]. This model has also been used for the discovery of genes and molecular pathways potentially involved in other fields of cellular physiology that can be relevant in cancer biology, such as resistance to cytotoxic agents [2]. One of these genes, pad1^+^, an essential gene in fission yeast, led to the cloning of human POH1 (Pad-One-Homologue1), which is a highly conserved functional homologue of fission yeast Pad1 and has been found to be a subunit of the human 26S proteasome [3]. Subsequent studies have shown that POH1 is a key player in the ubiquitin/proteasome pathway with deubiquitinase activity [4, 5] and a potentially interesting drug target for cancer therapies [6]. Here we give an overview of the studies on this protein over the past two decades, starting from knowledge acquired in basic research through functional and structural studies and discussing in vivo data that suggest a role of POH1 as a marker of poor prognosis and drug resistance in cancer. Finally, we will review recent data on compounds that target POH1 and their potential application in cancer treatment.

## POH1 and the ubiquitin/proteasome system

POH1 is a protein of 310 amino acids encoded by a 12-exon gene located on chromosome 2q24.2. It is ubiquitously expressed in human tissues and has a molecular weight of 34.6 kDa [3]. Its sequence is highly conserved through evolution from unicellular eukaryotes to human and contains in the N-terminal part a domain called MPN (for MPR1/Pad1/N-terminal) [7] with a catalytic site consisting of a conserved sequence called JAMM motif (JAMM = JAB1-MPN-Mov34). Two conserved histidines (H113 and H115) in this motif coordinate a zinc ion and POH1 acts as a metallo-protease [8]. It has deubiquitinase activity and cleaves isopeptide bonds between proteins and the polyubiquitin chain that tags substrate proteins before proteosomal degradation [4, 5]. POH1 is a subunit of the regulatory particle of the proteasome (19S proteasome) and is located in the outer part called lid subcomplex [9]. POH1 is also named Rpn11 (Regulatory particle subunit number 11) and the corresponding gene has been named *PSMD14* (Proteasome 26S subunit, non-ATPase 14) according to the HUGO Gene Nomenclature Committee.

The ubiquitin–proteasome system (UPS) is the most important pathway of intracellular protein degradation with a fundamental role in cellular homoeostasis [10]. The UPS is highly regulated and involves a system of tagging of protein substrates with the ubiquitin protein, which can form a chain by subsequent binding of additional ubiquitins. This process involves three classes of enzymes working in sequence that are classified as E1 (ubiquitin-activating enzymes), E2 (ubiquitin-conjugating enzymes) and E3 (ubiquitin ligases) [11]. Once a protein is labelled by a polyubiquitin chain, it can be degraded by the proteasome. The structure of the 26S proteasome has been described in details based on crystallographic studies both in yeast and in human [12, 13]. It is a multiprotein complex with a cylindrical core called the 20S proteasome, which is made of 28 subunits arranged in a pile of 4 rings of 7 subunits, with a narrow central channel in which the protein substrate is translocated and degraded [14, 15]. The outer rings are made of seven alpha-subunits and the inner rings by seven beta-subunits. Three of the beta-subunits contain the proteasome’s enzymatic active sites and each site has similar cleavage specificity to a known protease: beta-1 has caspase-like activity, beta-2 has trypsin-like activity and beta-5 has chymotrypsin-like activity. At each extremity of the 20S proteasome, another complex called the 19S proteasome or the regulatory particle of the proteasome is composed of 18 subunits arranged in 2 subcomplexes, a ring-shaped base of 9 subunits that is bound to the 20S proteasome and an outer part with the shape of a lid composed of 9 subunits [9]. The base of the 19S proteasome contains six subunits with ATPase activity (AAA-proteins), two non-ATPases (Rpn1 and Rpn2) and a receptor for ubiquitin (Rpn13). The lid of the 19S proteasome contains six structurally related proteins (PCI proteins) arranged in a horseshoe-like ring (PCI = *P*roteasome-*C*OP9-*I*nitiation factor3), a ubiquitin-receptor (Rpn10) and a heterodimer of structurally related proteins Rpn8 and Rpn11/POH1 [7].

Ubiquitination of protein substrates is a reversible process. On the one hand, specific E3 ubiquitin ligases act on various proteasome substrates and on the other specific proteolytic enzymes called deubiquitinases can remove ubiquitin tags from the substrates. There are five subclasses of deubiquitinases based on the type of Ub-protease domain [16]. Four subclasses are Cysteine proteases: ubiquitin-specific proteases (USP), ubiquitin carboxy-terminal hydrolases (UCH), Otubain proteases (OTU), Machado-Joseph disease proteases (MJD). The other subclass, to which POH1 belongs, are zinc-dependent metallo-proteases defined by the presence of the JAMM domain.

The proteasome can be associated with two deubiquitinases, USP14 and UCH37. In contrast, POH1 is the only deubiquitinase that is structurally linked to the proteasome and confers this catalytic activity to the complex [4, 5]. Its mode of function has been studied extensively and available evidence suggests that its activity is highly regulated and depends on the interaction with other proteasome subunits and on its conformational state [17, 18]. POH1 removes polyubiquitin chains from the substrates allowing their translocation into the 20S proteasome and their degradation by proteolytic enzymes of the core of the proteasome. Unlike USPs and UCHs that are involved in removal of single ubiquitins, POH1 has been shown to remove the full polyubiquitin chain by hydrolysing the isopeptide bond between the substrate and the C-terminus of the first ubiquitin. The presence of a deubiquitinating activity associated with the proteasome was known before the discovery of POH1 [19]. In 2002, two groups identified independently POH1 as the deubiquitinase located in the lid of the proteasome that was responsible for removal of polyubiquitin chains from substrates. These studies showed that the deubiquitination activity of POH1 was coupled to protein degradation and was essential for efficient substrate degradation. POH1 appeared to be a Zn^2+^-dependent metallo-protease and this activity was mapped to the consensus sequence EX(n)HS/THX[7]SXXD that further defined the so-called JAMM/MPN+ superfamily of metallo-proteases [16]. In this active site, a zinc ion is chelated to the two histidines and the aspartic residue of the JAMM/MPN+ sequence [20, 21]. The catalytic site is essential in *Saccharomyces cerevisiae* and mutation of histidine residues to alanine in the active site was lethal [4]. Subsequent structural studies on the Rpn11/Rpn8 dimer have shown that the catalytic groove of POH1 differs from that of other JAMM proteases given that the part of the sequence called Insert-2 does not interact with ubiquitin but rather with the non-ATPase subunit Rpn2 of the base with the result of anchoring POH1 within the complex [22, 23]. The anchoring of POH1 is supposed to stabilise the unstable catalytic loop of the protein [22].

**Fig. 1:**
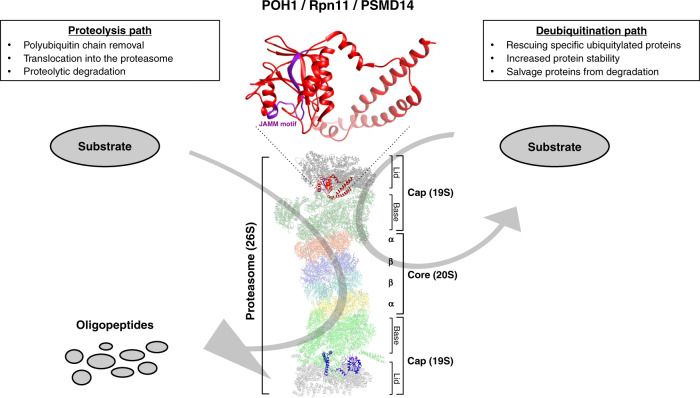
Structure of the 26S proteasome and position of POH1 (coloured in red) within the lid of the 19S regulatory particle. The 3D structure of POH1 within the proteasome is magnified and the position of the JAMM active site is coloured in purple. The figure was prepared with chimera (v1.15) [96] using the crystal structures downloaded from the protein data bank with code 5GJR [97].

In summary, POH1 has unique molecular features and is a crucial player within the proteasome where it acts as the only intrinsic deubiquitinase and has a peculiar catalytic motif in its active site. These discoveries have suggested that POH1 could become a novel target within the proteasome after the successful development for cancer treatment of proteasome inhibitors that target the chymotrypsin-like activity of the beta-5 subunits of the proteasome core [24, 25]. Aside from its direct contribution to substrate degradation through removal of the polyubiquitin chain that allows efficient substrate translocation into the proteasome, its deubiquitinase activity raised the question of its role in “proof-reading” ubiquitinated substrates and in regulating the balance between destruction and rescue of substrates [26]. In fact, several lines of evidence suggest that, by its deubiquitinase activity, POH1 might not only contribute to proteolytic degradation by the proteasome but also increase the stability of various ubiquitinated substrates [27–30] (Fig. 1).

## Proteasome inhibitors

The UPS was first identified and studied in the late ‘70s and early ‘80s [31] but it was not until the end of the ‘90s that its fundamental importance was recognised. Along with its physiological importance, it is now recognised that the UPS can be involved in multiple human diseases, including in particular neurodegenerative diseases and cancer [32, 33]. Inhibitors of the proteasome, such as lactacystin and MG132 that can block the chymotrypsin-like activity of the beta-5 subunit of the 20S proteasome, were used as laboratory tools before the application of proteasome inhibitors in the clinic. A boronate derivative of MG132, bortezomib, was the first proteasome inhibitor developed for clinical use. It is a potent inhibitor of the chymotrypsin-like activity of the beta-5 subunit with an IC_50_ of 7 nM. Early clinical trials revealed an important and unexpected therapeutic activity against multiple myeloma. Bortezomib was approved by the Food and Drug Administration (FDA) in 2003 for refractory multiple myeloma and subsequent trials confirmed that bortezomib could improve dramatically the outcome of this disease [34]. Bortezomib remains until now the cornerstone of multiple myeloma treatment and is used in first-line treatment for newly diagnosed multiple myeloma. Bortezomib was also licensed for the treatment of mantle-cell lymphoma and was effective in early clinical trials to treat other blood neoplasias for which, however, it was not further developed. In contrast, bortezomib had lower or absent activity in solid tumours in which it was tested. The reason for the specificity of bortezomib against multiple myeloma cells could be due to the inhibitory effect on the nuclear factor (NF)-kB pathway (by inhibiting proteasome degradation of the NF-kB inhibitor IkB) [35]. Another reason could be the important secretion of immunoglobulins by multiple myeloma cells, which makes them vulnerable to proteotoxic stress by the accumulation of misfolded proteins in case of proteasome inhibition [36]. Resistance to bortezomib can occur by several mechanisms. Mutations in the beta-5 subunits have been identified, as well as changes in proteasome subunit composition, along with off-target mechanisms like the expression of antiapoptotic proteins and changes in the transcriptome [37]. Another limitation of bortezomib is the frequent development of peripheral neuropathy, especially at high cumulative doses.

Carfilzomib was the first second-generation proteasome inhibitor to be developed. It has a structure based on the natural product epoxomicin and inhibits the beta-5 subunits with high potency (IC_50_ value of around 6 nM) and with an irreversible covalent bond with the active site. It was approved by the FDA in 2012 for relapsed/refractory multiple myeloma and has lower toxicity for peripheral nerves but higher cardiovascular toxicity compared to bortezomib [38, 39]. The third proteasome inhibitor approved for clinical use, ixazomib, has been approved by the FDA in 2015. It has a boronic acid–base structure similar to bortezomib but is administered orally as a prodrug that hydrolyses to form the active inhibitor. It is also a reversible inhibitor and has a much faster off-rate for proteasome binding that allows for larger tissue distribution compared to bortezomib [40]. Other direct proteasome inhibitor targeting the beta-5 subunits have been studied but none of them has reached clinical development [41]. VLX1570, an inhibitor of USP14, an extrinsic deubiquitinase that can be associated with the proteasome, has entered a phase 1 clinical trial but development was stopped due to toxicity [42]. Other strategies of targeting specific molecules of the UPS upstream from the proteasome (modulators of E1, E2 and E3 enzymes and p97/VCP involved in endoplasmic reticulum-associated protein degradation) are being pursued and most of them are in the pre-clinical phase [41].

## The family of MPN-domain containing proteins and JAMM/MPN deubiquitinases

**Fig. 2:**
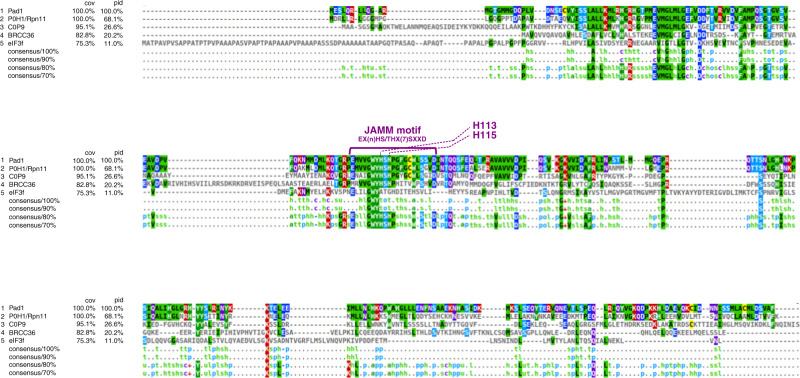
Protein sequence alignment of *S. pombe* Pad1, human POH1 and other MPN proteins associated with multiprotein complexes CSN5, BRCC36 and eIF3f. The multiple sequence alignment was made with EMBL-EBI’s tools Clustal Omega and MView [98].

The first hint that POH1 could be a proteasome subunit came from the similarity between the sequence of the POH1 protein and the proteasome subunit Mov34 (currently known as Rpn8) that share the MPN domain. It was hypothesised that POH1 could also be a subunit of the proteasome like Rpn8 and immunoblot analysis of human purified proteasomes showed that POH1 was part of the regulatory particle of the human proteasome [3]. The Pad1 protein in *S. pombe* was also shown to be a subunit of the 26S proteasome [43]. Structural studies on the 26S proteasome in *S. cerevisiae* found that the POH1 and Rpn8 homologues in budding yeast were the only two subunits of the lid of the proteasome containing an MPN domain that was located in the N-terminal part of the protein [7]. In addition to the demonstration of the deubiquitinase activity of POH1 [4, 5] and the mapping of the active site to the JAMM sequence motif [8], it was shown that the CSN5 protein of the signalosome was a JAMM isopeptidase [44]. A genomic approach with bioinformatics analysis identified 14 human proteins containing MPN domains [16]. Subsequent biochemical and structural studies (reviewed in [45]) have found that eight of them are part of multiprotein complexes such as the 19S proteasome, the COP9 signalosome, the RAP80 complex (a BRCA1/BRCA2-containing complex) and eIF3 (for eukaryotic translation initiation factor 3). Members of this super-family can be classified into two categories: those containing a catalytically zinc-dependent metallo-isopeptidase motif, also called JAMM/MPN+ and those lacking this catalytic motif. Each of the above multiprotein complexes contains a single heterodimer of MPN proteins, usually with one catalytically active and one inactive monomer (POH1-Rpn8 in the proteasome, CSN5-CSN6 in the signalosome and BRCC36-Abraxas in the RAP80 complex). The catalytically inactive protein has mainly a structural role and the active partner carries the enzymatic function [45] (Table 1). The catalytically active monomers POH1, CSN5 and BRCC36 have a certain degree of sequence similarity and contain the JAMM active site (Fig. 2). In the eIF3 complex, the eIF3f and eIF3h subunits are MPN-domain proteins and eIF3f has been shown to have deubiquitinase activity on the transcription factor Notch [46], although it has an incomplete JAMM active site. Recent works on the structure of JAMM proteins of the archebacterium *Pyrococcus furiosus* have mapped the interface between the parts of the MPN dimer and have studied the influence of the conformational state on the catalytic activity [47]. With regard to POH1, there is evidence that the isolated MPN heterodimer POH1-Rpn8 is more active in vitro than the 26S proteasome lid, which could lock Rpn11 in an inhibition state through Rpn5 [17].

**Table 1:** Pairs of MPN-domain proteins forming dimers within multiprotein complexes and biological pathways in which they are involved.

JAMM/MPN+ protein (catalytically active)	MPN protein (catalytically inactive)	Complex	Biological pathway
POH1/Rpn11	Mov34/Rpn8	Proteasome lid	Protein degradation
CSN5	CSN6	COP9 signalosome	Protein degradation/signalling
eIF3f^a^	eIF3h	eIF3	Protein biosynthesis
BRCC36	Abraxas	RAP80	DNA damage

^a^eIF3f does not have a complete JAMM sequence motif, in contrast to the other JAMM/MPN+ proteins (see also Fig. 2).

It is known that the 19S complex can exist in an unbound form, although its main function is considered to be the activation and regulation of the 20S proteasome upon binding and forming 26S proteasomes [48]. Within the 19S complex, the lid subcomplex is located at the outer extremity of the 26S proteasome and has a striking structural similarity with the COP9 signalosome. Both complexes contain an MPN dimer (CSN5-CSN6 in the COP9 signalosome and POH1-Rpn8 in the proteasome lid) and six PCI-domain proteins. The PCI proteins oligomerise to form a horseshoe-like structure and the MPN-domain heterodimer is situated on the top of this structure [7]. The COP9 signalosome (CSN) has been shown to be a signalling platform and a dynamic and heterogeneous structure [49]. CSN5, the paralog of POH1 in the signalosome is an intrinsic JAMM/MPN isopeptidase within the CSN and cleaves the ubiquitin-like protein Nedd8 from Cullin within the SCF ubiquitin ligases [44, 50]. There is evidence that the COP9 signalosome interacts with the 26S proteasome [51] and it has been hypothesised that, under certain circumstances, the COP9 signalosome might substitute for or compete with the lid of the 19S proteasome [49].

## Biological processes influenced by POH1

Given its fundamental molecular function as the obligate deubiquitinase of the 19S regulatory particle of the proteasome, not surprisingly POH1 can be linked to a variety of biological processes in which the UPS is involved, such as cell cycle progression, signal transduction, protein quality control and differentiation. However, some data suggest that POH1 and the 19S complex could also have functions that are independent of the 20S proteasome.

First, several lines of evidence from work in *S. cerevisiae* suggest that the carboxyl-terminal domain of Rpn11 has a role in the maintenance of mitochondrial structure and function [52–55]. This function might be related to a fraction of the protein that is stable and outside the proteasome [53]. Experiments of cellular subfractionation have found a small portion of the Rpn11 protein associated with mitochondria, while most of the protein was found in the cytosolic fraction [52].

Studies in tumour cell lines have found highly variable protein levels across cell lines and differences of the ratio of POH1 to 20S proteasome subunits [56]. These results support the hypothesis that a fraction of POH1 exists in a form not permanently associated with the 26S proteasome. One hypothesis to be tested is that cancer cells with aberrant amounts of POH1 protein might contain an excess of POH1 in the form of free MPN dimers or lid subcomplexes [47, 57].

Second, a clinically relevant field in which POH1 might have a role independently of the full 26S proteasome is DNA repair. In the response to DNA double-strand breaks, experiments in cells exposed to hydroxyurea followed by small interfering RNA (siRNA) screen identified POH1 as an enzyme required to reduce DNA–Ubiquitin conjugates [58]. It was also shown that POH1 co-operates with BRCA1 in DNA double-strand break repair in G2 phase cells and promotes homologous recombination [59].

Third, the UPS has been shown to play an important role in the regulation of pluripotency of stem cells, and in particular POH1 expression has been found to be essential for maintenance of embryonic stem cells pluripotency and self-renewal [60]. Pluripotent stem cells express high levels of POH1, whereas the expression is downregulated as the cells differentiate. POH1 knockdown in stem cells leads to decrease of Oct4 expression, a key positive regulator of self-renewal [60]. It is known that both haematological and solid tumours contain a fraction of cancer stem cells that share general features of stem cells [61]. These cells are considered chemoresistant clones that can persist after treatment with cytotoxic drugs and ultimately lead to relapse or progressive disease [62]. Recent work in colorectal cancer cell lines and in a xenograft model have found that POH1 facilitates tumour growth and cancer stemness/chemoresistance by deubiquitination of the ALK2 receptor, which in turn activates the bone morphogenetic 6 (BMP6) signalling pathway [30].

Fourth, some data suggest an influence of POH1 in the phenomenon of “epithelial-to-mesenchymal transition” (EMT). Experiments of knockdown of POH1 by siRNA in cancer cell lines from breast cancer, melanoma and oesophageal cancer have found a decrease of features of EMT transition [29, 63, 64]. EMT is known to be associated with tumour progression, metastasis and drug resistance [65].

In summary, by its key role within the proteasome, POH1 is involved in many intracellular pathways that are potentially relevant for cancer biology. Some of the processes that are influenced by POH1 could be mediated by factors not affected by the main proteolytic activity of the 26S proteasome and involving the sole 19S regulatory particle or other POH1-containing subcomplexes (the proteasome lid or the POH1-Rpn8 dimer), although experimental proof of this concept is lacking due to the difficulty of separating proteasome binding from POH1 function. Several downstream mechanisms of POH1 upregulation are associated with a selective growth advantage for cancer cells and are summarised in Table 2.

**Table 2:** Potential mechanisms conferring selective growth advantage or chemoresistance in POH1-overexpressing cancer cells.

Mechanism	Reference
c-JUN stabilisation and AP-1-mediated transcription	Nabhan and Ribeiro [27]
E2F1 stabilisation and downstream prosurvival signals	Wang et al. [28]
	Zhi et al. [90]
	Jing et al. [87]
Increased DNA repair after double-strand breaks	Butler et al. [58]
	Kakarougkas et al. [59]
Stabilisation of the BMP type 1 receptor ALK2	Seo et al. [30]
Stabilisation of SNAIL and SLUG, favouring EMT	Luo et al. [63]
	Yokoyama et al. [29]
	Jing et al. [64]

## POH1 and activation of AP-1 transcription factors

AP-1 transcription factors have been implicated in tumorigenesis [66] and in tumour multidrug resistance [67–69]. Activation of this pathway has been shown to be one of the downstream effects of POH1 overexpression in human cells and Pad1 overexpression in fission yeast [3, 27, 70]. In fission yeast, several independent mechanisms lead to a phenotype of multidrug resistance that depends on the AP-1 transcription factor Pap1. The multidrug resistance phenotype induced upon Pad1 and POH1 overexpression in *S. pombe* is dependent on the presence of the gene encoding Pap1 [3]. Pap1 is involved in the oxidative stress response and is regulated at various levels. First, Pap1 is subject to nucleocytoplasmic shuttling and oxidative stress induces a conformational change that prevents its interaction with the exportin Crm1, which in turn results in its nuclear accumulation [71]. As a result, several mutations in the crm1^+^ gene also confer a pap1^+^*-*dependent multidrug resistance phenotype, by defective Crm1 function and Pap1 nuclear accumulation [72, 73]. Second, Pap1 activity can be influenced in fission yeast at the translational level, as overexpression of Int6, a component of the eIF3 complex and the homologue of the mammalian oncoprotein Int6, leads to an increase of Pap1-responsive mRNAs [74, 75]. Activation of AP-1-dependent transcription by Int6 is associated with upregulation of 67 genes containing AP-1 consensus binding sites in their upstream regulatory sequences. Many of these fission yeast genes encode proteins known to have a role not only in detoxification or membrane transporters but also in signal transduction, regulation of transcription and differentiation [75]. Based on the studies in *S. pombe*, where the Pad1 and POH1 overexpression phenotype was dependent upon the expression of the pap1^+^ gene, it was hypothesised that POH1 overexpression could decrease proteasome degradation of AP-1 transcription factors and lead to increased transactivation of AP-1-responsive genes. Experiments in the flatworm *Schistosoma mansoni*, where the POH1 protein is 78% identical to human POH1, showed that this was indeed the case [76]. Further work in human HEK293 cells confirmed that induced overexpression of the POH1 protein leads to a stabilisation of c-JUN, mediated by a decrease of its level of ubiquitination, an accumulation of the protein and an increase in AP-1-mediated gene expression. This effect appeared to be selective for c-JUN as it was not observed on other proteasome substrate such as p27/kip1. This was considered related to the deubiquitinase activity of POH1 as it was reduced by mutation of the Cys-120 site within the active JAMM motif [27]. Other experiments obtained in fission yeast with a screen for mutants resistant for the microtubule poison methyl benzimidazol-2-yl-carbamate (MBC) have found several temperature-sensitive mutants with mutations in genes coding for various subunits of the proteasome, including pad1^+^, and for genes involved in the ubiquitination of Pap1, that are multidrug resistant due to stabilisation of Pap1 [77]. Therefore, in fission yeast a similar but non-identical phenotype of multidrug resistance depending on the AP-1 transcription factor Pap1 is observed both by mutations in several proteasome subunits [77] and by overexpression of pad1^+^ [70]. In mammalian cells, overexpression of POH1 in COS7 cells leads to moderate resistance to various anticancer agents [3, 56] and overexpression in HEK293 cells leads to a decrease of c-JUN ubiquitination, with an increase in its stability and in transcription of AP-1-responsive genes [27]. Findings in fission yeast could also be explained by Pad1 overexpression interfering with proteasome assembly and mimicking loss of function; however, the overexpressed POH1 protein in HEK293 cells was shown to incorporate into the proteasome without interfering with proteasome assembly [27]. Therefore, available evidence is consistent with the dual function of POH1, which contributes to proteasome degradation activity (with defects leading to accumulation of their substrates including Pap1 in fission yeast) and acts as a deubiquitinase that might salvage specific substrates from degradation [26], for instance c-JUN or other substrates in mammalian cells [27, 29, 30, 63, 64] (Fig. 1).

## POH1 as a drug-resistance marker

POH1 has been cloned in 1996 by hybridisation to a probe generated by nested PCR with degenerate primers and was shown to complement deletion of the pad1^+^ gene in *S. pombe* [3] Pad1^+^ was first isolated as a multicopy plasmid conferring resistance to staurosporine [70]. It is an essential gene as pad1-deleted strain are not viable. Overexpression of pad1^+^ in *S. pombe* induced a phenotype of resistance not only to staurosporine but also to multiple other toxic agents, such as brefeldin A, caffeine and cycloheximide. The human POH1 gene encodes for a protein with a high degree of sequence similarity with fission yeast Pad1 (Fig. 2), is a fully functional homologue of Pad1 and induces by overexpression the same phenotype of pleiotropic drug resistance in *S. pombe*.

To assess whether POH1 could be involved in resistance to cytotoxic insults and in particular to anticancer agents in mammalian cells, experiments of POH1 overexpression by transfection in mammalian cells were performed. Results showed a phenotype of resistance not only against staurosporine but also against anticancer drugs, such as paclitaxel, doxorubicin, cisplatin, melphalan and vinblastine [3, 56]. The decrease in drug sensitivity was quantitatively mild with increases of the IC_50_ of about 2.0/2.5-fold. The multidrug resistance phenotype appeared to be independent from the expression of the membrane pump P-glycoprotein 170, as the efflux of rhodamine, a substrate of P-glycoprotein, was not influenced by POH1 overexpression and POH1 overexpression also conferred increased resistance to physical agents like ultraviolet (UV) radiation [3]. A role for POH1 in mediating resistance to UV radiation has also been reported in budding yeast, where several strains containing mutations of Rpn11 had higher sensitivity to UV radiation compared to wild-type strains and Rpn11 was required for the UV resistance pathway dependent on the AP-1-like transcription factor Gcn4 [78].

Was this POH1-related pathway also operating in biological systems under physiological conditions? And if yes, could this be a mechanism used by cancer cells to resist cytotoxic drugs? Work on marine organisms such as the marine sponges *Geodia cydonium* that are exposed to a diverse range of environmental xenobiotics showed that POH1 expression could be induced in the laboratory after few days of exposure to staurosporine and paclitaxel. Field experiments in marine areas with variable pollution loads showed that the expression of POH1 was higher in sponges exposed to polluted water than in those living in less polluted water, suggesting that POH1 expression could be an inducible mechanism operating in nature and could act as an indicator of water pollution [79]. In cancer cells, the content of the POH1 protein has been measured in cell lysates of the panel of 60 cancer cell lines used by the U.S. NCI for drug screening [80]. It was found that POH1 protein levels measured by western blot and densitometry vary over a wide range within all nine tumour types of the panel and are significantly higher in cell lines with intrinsic resistance to anticancer drugs [81].

More recently, the ratio between the full 26S complex and the 20S proteasome has been found to be increased upon oncogenic transformation of human and mouse cells and transformed cells have been shown to be dependent on high levels of 26S proteasomes [82]. In this work, drug-resistant cell lines were more vulnerable to depletion of 26S proteasomes in comparison to non-drug-resistant tumour cell lines, suggesting that multidrug resistant cancer cells could be dependent on high 26S proteasome levels [82].

In summary, the mechanisms linking POH1 to drug resistance remain not fully elucidated. However, a large body of data suggest that POH1 might act as a positive regulator of AP-1-regulated genes leading to a drug resistance phenotype [3, 27, 67, 69, 75] and recent work suggests that it could also be involved in the regulation of transcriptional programmes associated with cancer stemness [30] and EMT [29, 63, 64].

## Translational studies on POH1 in human cancer

Studies on the expression of POH1 in human cancer cell lines and/or tumour specimens have been published in multiple myeloma [83], breast cancer [63], hepatocellular carcinoma [84, 85], colorectal cancer [30, 85], prostate cancer [86], oesophageal carcinoma [64, 85], head and neck squamous cell carcinoma [87], lung adenocarcinoma [88], melanoma [29] and osteosarcoma [89]. Most of these studies (summarised in Table 3) included both an analysis of clinical specimens with an assessment of the prognostic value of POH1 expression and experiments on tumour cell lines or human tumour xenografts with the aim of elucidating the downstream effects of POH1 aberrant expression and/or POH1 knockdown.

**Table 3:** Summary of translational studies on POH1 in tumour cell lines, tumour specimens and xenograft models.

Tumour type	Experimental methods	Observed phenotype	Proposed mechanism	Reference
Multiple myeloma	Tumour specimens (bone marrow)	POH1 expression in Tumour > Normal plasma cells		Song et al. [83]
	Specimens of MGUS, smouldering myeloma and active myeloma	POH1 expression correlates with disease progression		
	Patients’ population receiving uniform treatment	Higher gene expression associated with shorter survival		
	Tumour cell lines POH1 siRNA knockdown and treatment with *O*-phenanthroline	Decreased tumour cell viability Overcomes bortezomib resistance Synergism with Dexametasone, Lenalidomide and Pomalidomide	Block of proteasome function, activation of caspase cascade and endoplasmic stress response	
	Human xenograft model *O*-phenanthroline treatment	Reduces progression of tumour growth and prolongs survival in mice		
Breast cancer	Tumour specimens and adjacent tissue (immunohistochemistry)	POH1 expression in Tu > Normal		Luo et al. [63]
	Treated population	Higher expression associated with shorter survival		
	Tumour cell lines POH1 siRNA knockdown	↓EMT transition ↓cell proliferation ↑G0/G1 arrest ↑apoptosis	↓SLUG ↓SNAIL	
	Tumour cell lines POH1 overexpression	↑cell growth ↓apoptosis		
Prostate carcinoma	Prostate samples, cancer and normal tissue (immunohistochemistry)	POH1 expression in Tu > Normal and in Gleason score ≥4 + 3 higher than in Gleason score ≤3 + 4		Yu et al. [86]
	Tumour cell lines POH1 siRNA knockdown Capzimin treatment	↓clonogenicity ↓growth	Cell cycle arrest, ↓cyclinD1, ↓phospho-RB	
	Gene set enrichment analysis of the TCGA and SU2C/PCF data set of expression profiles		Correlation between POH1 expression and E2F target genes	
	Tumour cell lines and xenografts of androgen-dependent and castration-resistant prostate cancer	Increased effect of docetaxel and androgen deprivation (Enzalutamide) by Capzimin treatment		
Oesophageal cancer (EC) Hepatocarcinoma (HCC) Colorectal cancer (CRC)	Immunohistochemistry on tumour specimens	Higher expression in tumour than in normal adjacent tissue		Wang et al. [85]
		High POH1 expression correlates with ↓overall survival in CRC and HCC		
	Tumour cell lines and tumour xenografts	↑apoptosis by POH1 knockdown (siRNA)	↓p53 degradation ↓Bim degradation	
Melanoma	Melanoma cell lines siRNA screening of 97 DUBs and focus on POH1	Decreased migration by POH1 knockdown	↓TGF-beta signalling ↓SLUG	Yokoyama et al. [29]
		Decreased proliferation by POH1 knockdown	↑p21 (mRNA) ↑p27 (protein, not mRNA)	
Lung adenocarcinoma	Immunohistochemistry on tumour specimens	Higher expression in tumour than in normal adjacent tissue High POH1 expression correlates with ↓overall survival and higher TNM stage		Zhang et al. [88]
	Tumour cell lines POH1 siRNA knockdown	↑apoptosis ↓growth, ↑senescence	↑p21 stability by ↓ degradation	
Oesophageal cancer (squamous, ESCC)	Mouse model of induced ESCC	POH1↑ in the transition from normal tissue to dysplasia and tumour		Jing et al. [64]
	Population database	High POH1 expression correlates with ↓overall survival		
	Tumour cell lines Thiolutin treatment	↓migration ↓invasiveness ↑sensitivity to cisplatin	↓SNAIL	
Head and neck squamous carcinoma (HNSCC)	Immunohistochemistry on tumour specimens	Higher expression in tumour than in normal adjacent tissue High POH1 expression correlates with worse prognosis		Jing et al. [87]
	Mouse model of induced HNSCC	Increased POH1 upon tumorigenesis	↑E2F1, ↑Akt pathway→↑SOX2	
	Tumour cell lines Loss of function assays and thiolutin treatment	↓growth, ↓stemness, ↓chemoresistance to cisplatin		
Osteosarcoma	Bioinformatics (differentially expressed genes)	POH1 top gene among upregulated genes ↑POH1 = ↓prognosis	10 hub genes encoding ribosomal proteins by protein–protein interaction network	Gong and Wei [89]

In multiple myeloma, POH1 gene expression was found to correlate in large data sets of newly diagnosed patients with decreased overall survival after uniform treatment. Interestingly, when analysing POH1 expression in samples from normal individuals and from patients with monoclonal gammopathy of unknown significance, smouldering multiple myeloma and overt myeloma, expression of POH1 correlated with progression of the disease, suggesting a contribution of POH1 in the pathogenesis of the disease [83]. In breast cancer, POH1 protein expression assessed by immunohistochemistry was found to be higher in tumour tissue than in adjacent normal tissue and to correlate by retrospective analysis with worse prognosis in terms of overall survival both in the population of 142 cases and in a validation reference population. The same group showed with experiments on breast cancer cell lines that POH1 knockdown by siRNA not only decreased cell proliferation but also decreased the level of markers of EMT, such as SLUG and SNAIL [63]. In the prostate, POH1 expression was found to be higher in human tumour samples than in normal tissue, and among tumours, the expression of POH1 correlated with the Gleason score [86]. Similarly, overexpression of POH1 in tumour tissue compared to adjacent normal tissue was demonstrated in oesophageal cancer, hepatocellular carcinoma, colorectal cancer and lung adenocarcinoma, with a worse prognosis in terms of overall survival for tumours with high POH1 versus low POH1 [84, 85, 88]. In hepatocellular carcinoma, it was also reported that POH1 expression at the protein level correlates with a molecular signature associated with metastatic potential in vitro and in vivo, and it was suggested that this might be mediated by the effect of POH1 on the transforming growth factor (TGF)-beta signalling pathway that is dysregulated in the progression of hepatocarcinoma [84]. In oesophageal squamous cell carcinoma, another group reported recently that POH1 was upregulated in the transition from normal tissue to epithelium dysplasia and carcinoma both in a murine model of chemically induced oesophageal carcinoma and in The Cancer Genome Atlas database [64]. In this work, POH1 levels were found to be positively correlated with the expression of SNAIL, a marker of EMT associated with metastatic potential. A similar observation has been reported in melanoma cell lines, where POH1 knockdown by siRNA resulted both in decreased proliferation and migration, with a concomitant decrease of the EMT marker SLUG [29]. In head and neck squamous cell carcinoma (HNSCC), POH1 was found to be associated with tumorigenesis using a murine HNSCC model and in vitro and in vivo experiments showed that POH1 depletion reduced HNSCC growth, chemoresistance and stemness [87].

In summary, translational studies show that POH1 expression is increased at the protein level in comparison with normal tissues not only in multiple myeloma but also across many frequent solid tumour types, including tumours with known intrinsic chemoresistance such as hepatocarcinoma and melanoma. Available evidence supports a correlation with more aggressive features and worse prognosis and experimental works on tumour cell lines [63, 64, 83–90] and in xenograft models [29, 64] have shown that targeting POH1 might have a profound anticancer effect.

## Pharmacological targeting of POH1

An explanation of the sensitivity of multiple myeloma cells to proteasome inhibitors is that they exhibit a lower threshold for induction of a lethal “unfolded protein response” (UPR) [91], because of their physiological production of large quantities of antibody proteins [36, 92]. This is the basis of the proteotoxic crisis model, which postulates that due to higher dependence of cancer cells in comparison to normal cells on mechanisms of protein quality control, targeting other components of the UPS might lead to new generally active cancer therapies [25]. POH1 is a particularly attractive target because its deubiquitination activity is coupled to substrate degradation [4, 5], its role within the 19S regulatory particle is unique [26] and its active site can become a pharmacological target [6, 8].

**Fig. 3:**
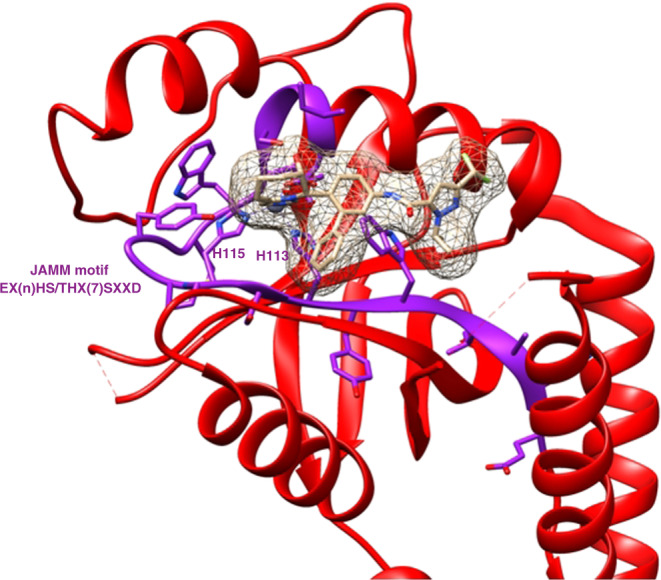
3D reconstruction of the interaction between capzimin and POH1 active site. The figure was prepared with chimera (v1.15) [96] using the crystal structures downloaded from the protein data bank with code 5JOG [99].

The first proof that POH1 could be targeted was provided by a group at the Dana Farber Cancer Institute that showed in 2017 that pharmacologic inhibition of POH1 with *O*-phenanthroline blocked proteasome function, had an anti-myeloma effect in human xenografts and, similarly to proteasome inhibitors in the clinic, was synergistic with the anti-myeloma activity of lenalidomide, pomalidomide and dexametasone [83]. This compound is a non-specific inhibitor of various metallo-isopeptidases and was not developed further. Another group at Caltech performed a search for POH1 inhibitors by establishing an assay that measures specifically the deubiquitinating activity of POH1 [6]. By screening both a focussed library of metal-binding pharmacophores and a vast library of 330,000 compounds in the National Institute of Health Molecular Libraries Small Molecule Repository, they identified quinoline 8-thiol (8TQ) and showed that 8TQ inhibited POH1 by binding to the catalytic Zn^2+^ ion. They subsequently improved the potency of 8TQ by an approach based on structure–activity relationship and identified that capzimin, a molecule that binds the catalytic site of POH1 (Fig. 3), has greatly enhanced selectivity for POH1 over other JAMM metalloenzymes and sevenfold more potency towards POH1 (IC_50_ = 0.34 mM) [6]. Capzimin was screened against the NCI panel of 60 cancer cell lines and exhibited a potent growth inhibition effect both on leukaemia cells and solid tumours and induction of cell death by apoptosis.

**Fig. 4:**
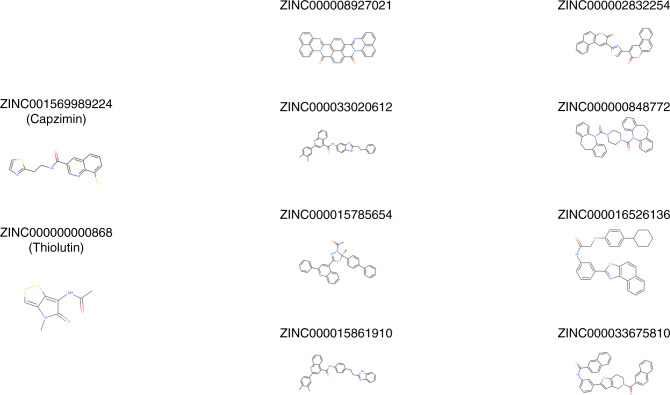
Chemical structures of capzimin, thiolutin and other compounds with predicted POH1-inhibitory activity based on virtual screening. Structures have been downloaded from the ZINC database [100].

Other compounds able to inhibit POH1, as well as other proteins of the MPN family have been described [93, 94]. These are naturally occurring compounds that are less potent and specific but could serve as lead compounds to guide the synthesis of more potent drugs. The first family of compounds, the epidithioldiketopiperazines, are related to an *Aspergillus fumigatus* toxin and have been shown to inhibit the catalytic active site Zn^2+^ of POH1. In particular, two molecules of this family have been identified with fewer non-specific effects and named SOP6 and SOP11. Another example is thiolutin, a compound belonging to the class of dithiolpyrrolones, which are antibiotics synthesised by actinomycetes and proteobacteria. Thiolutin was shown to be an inhibitor of POH1 with an IC_50_ = 0.53 mM. Holomycin, a methyl derivative of thiolutin, was even more efficient (IC_50_ = 0.18 mM). Interestingly, thiolutin was recently found in a model of oesophageal carcinoma to suppress motility and stemness and to increase sensitivity to cisplatin both in vitro and in vivo, by impairing the interaction between POH1 and SNAIL, a substrate of the proteasome with a role in EMT [64]. New methods of drug discovery based on large-scale virtual screening and classification by neural networks are increasingly applied, and by molecular simulations, eight structures of Rpn11 inhibitors have been recently selected [95] (Fig. 4).

## Conclusion, open questions and future perspectives

POH1 has a crucial role in cellular homoeostasis of human cells, mediated by its regulatory role as an intrinsic deubiquitinase within the proteasome [4, 5]. Some data suggest that POH1 might have other functions, not directly linked to the proteolytic activity of the 26S proteasome, such as a role in maintenance of mitochondrial structure [52], in response to DNA damage [58, 59] or in self-renewal of stem cells [60]. In these processes, POH1 could be involved as part of proteasome subcomplexes (the 19S regulatory particle, the lid subcomplex or a POH1-Rpn8 MPN dimer).

Ectopic overexpression of POH1 has been shown to decrease sensitivity to cytotoxic agents in mammalian cells [3, 56]. Overexpression of the POH1 protein also occurs in cancer cells of different tissues of origin both in cultured cell lines and in tissue specimens [63, 64, 83–90]. In translational studies performed in many tumour types, POH1 expression has been reported to increase in tumorigenesis models upon transition from pre-neoplastic to neoplastic tissue [64, 87], to correlate with higher tumour grade in established neoplasias [83, 86] and to be associated with decreased overall survival of patients [63, 84, 85, 88, 89].

More studies are needed to better understand the mechanisms and the significance of POH1 deregulation in cancer. It will be of utmost importance to clarify to what extent POH1 is overexpressed in tumours relative to normal tissue and whether this is linked to a general increase of 26S proteasome complexes or to an increase of POH1-containing subcomplexes or dimers.

Transcriptome studies should inform what type of molecular signatures are related to POH1 expression. A few studies have addressed this aspect in hepatocarcinoma [84], prostate cancer [86] and melanoma [29], with results suggesting an influence of POH1 on E2F target genes and on TGF-beta signalling. More extensive studies in multiple tumour types and in particular on drug-resistant cancers are needed.

One potential explanation for POH1 overexpression in tumours is that cancer cells require an upregulation of the mechanisms of protein quality control to cope with the increased amount of misfolded and aberrant proteins. If this is the case, targeting POH1 might lead to the so-called proteotoxic crisis due to the break of the proteostasis balance [25] similarly to what is observed in multiple myeloma treated by proteasome beta-5-inhibitors [36, 92]. In that model, POH1 inhibitors might bring added value to existing proteasome inhibitors and be useful in multiple myeloma, for instance, in case of resistance to proteasome inhibitors due to mutations of their target (the beta-5 subunit). Indeed, in vitro data show a higher inhibitory effect of capzimin in comparison to bortezomib in such a context [6].

In addition, data emerging from translational research strongly suggest that POH1 overexpression confers a selective advantage for the acquisition of the malignant and metastatic phenotype in cancer cells and is associated with chemoresistance [29, 63, 64, 87]. Therefore, POH1 could represent an important novel therapeutic target not only for multiple myeloma but also for solid tumours, including tumour types for which only few therapeutic options are currently available.

Efforts to develop POH1 inhibitors are ongoing and several selective POH1 inhibitors have been reported, even though none of them has been developed up to clinical assays in human so far [6, 93, 94]. The progress in studies on the structure of the proteasome and on the conformational states of the various subunits [18] and the introduction of new technologies for drug screening such as virtual screening will certainly help in the identification of more potent and specific inhibitors in the future. Very recently, at least eight new structures of potential POH1 inhibitors have been reported by such an approach [95].

Given the homoeostatic role of the proteasome and the expression of POH1 in normal cells and in all human tissues, it will be crucial to test POH1 inhibitors in the appropriate context in order to have a favourable therapeutic index. This could be done in multiple myeloma, especially in case of resistance to existing proteasome inhibitors, or in solid tumours selected based on the level of POH1 overexpression. For this purpose, a scoring system for assessing the level of expression by immunohistochemistry has been proposed [85] but should be further developed and validated. To overcome the risks of significant side effects in normal tissues by systemic delivery of POH1 inhibitors, strategies of selective delivery of the POH1 inhibitor to the cancer cell through antibody–drug conjugates or local delivery would also be important. Finally, based on the correlation between POH1 levels and resistance to several cytotoxic drugs, POH1 inhibitors should be tested not only as single agents but also in combination with existing anticancer agents with the aim of overcoming drug resistance. Recent work in mice supports this approach; local injection of the POH1 inhibitor thiolutin increased sensitivity to cisplatin in a xenograft model of oesophageal cancer [64].

## Data Availability

Not applicable.
